# Lysyl Oxidase Is Downregulated by the EWS/FLI1 Oncoprotein and Its Propeptide Domain Displays Tumor Supressor Activities in Ewing Sarcoma Cells

**DOI:** 10.1371/journal.pone.0066281

**Published:** 2013-06-04

**Authors:** Noelia Agra, Florencia Cidre, Laura García-García, Juan de la Parra, Javier Alonso

**Affiliations:** Unidad de Tumores Sólidos Infantiles, Área de Genética Humana, Instituto de Investigación de Enfermedades Raras, Instituto de Salud Carlos III, Majadahonda, Madrid, Spain; University of Navarra, Spain

## Abstract

Ewing sarcoma is the second most common bone malignancy in children and young adults. It is driven by oncogenic fusion proteins (i.e. EWS/FLI1) acting as aberrant transcription factors that upregulate and downregulate target genes, leading to cellular transformation. Thus, identificating these target genes and understanding their contribution to Ewing sarcoma tumorigenesis are key for the development of new therapeutic strategies. In this study we show that lysyl oxidase (LOX), an enzyme involved in maintaining structural integrity of the extracellular matrix, is downregulated by the EWS/FLI1 oncoprotein and in consequence it is not expressed in Ewing sarcoma cells and primary tumors. Using a doxycycline inducible system to restore LOX expression in an Ewing sarcoma derived cell line, we showed that LOX displays tumor suppressor activities. Interestingly, we showed that the tumor suppressor activity resides in the propeptide domain of LOX (LOX-PP), an N-terminal domain produced by proteolytic cleavage during the physiological processing of LOX. Expression of LOX-PP reduced cell proliferation, cell migration, anchorage-independent growth in soft agar and formation of tumors in immunodeficient mice. By contrast, the C-terminal domain of LOX, which contains the enzymatic activity, had the opposite effects, corroborating that the tumor suppressor activity of LOX is mediated exclusively by its propeptide domain. Finally, we showed that LOX-PP inhibits ERK/MAPK signalling pathway, and that many pathways involved in cell cycle progression were significantly deregulated by LOX-PP, providing a mechanistic explanation to the cell proliferation inhibition observed upon LOX-PP expression. In summary, our observations indicate that deregulation of the LOX gene participates in Ewing sarcoma development and identify LOX-PP as a new therapeutic target for one of the most aggressive paediatric malignancies. These findings suggest that therapeutic strategies based on the administration of LOX propeptide or functional analogues could be useful for the treatment of this devastating paediatric cancer.

## Introduction

Ewing sarcoma is an aggressive neoplasm that mainly affects child and young adults in the first and second decade of life. It mainly occurs in bones although a small percentage of these tumors also arise in soft tissues. Even though the overall survival rates have significantly risen in the last decades, an elevated percentage of these tumors are refractory to conventional chemo- and radiotherapy, making more necessary the development of new therapeutic strategies (reviewed in [1]). The development of new therapeutic strategies will only be possible through a better knowledge of the molecular mechanisms that govern the process of malignant transformation in these tumors.

The molecular hallmark of Ewing sarcoma is the presence of chromosomal translocations that generate fusion proteins with aberrant transcriptional activities. The most common of these translocations, observed in approximately 85% of the cases, is t(11;22) that fuse the EWS gene to the FLI1 transcription factor resulting in the EWS/FLI1 fusion protein. Other fusion proteins involving the EWS gene (and less frequently other related genes) and other transcription factors of the ets family have been described in the remainder cases. During the last years, important efforts have been made to identify gene targets of the EWS/FLI1 oncoprotein in Ewing sarcoma cells (reviewed in [2]–[6]). Many of these target genes have been shown to regulate cell proliferation, invasiveness, metastasis or responsiveness to oxidative stress in Ewing sarcoma cells (reviews above and [7])

Cellular models engineered to silence EWS/FLI1 expression by means of RNA interference have been very useful for the identification and characterization of relevant downstream targets of EWS/FLI1 [8]–[19]. Particularly, inducible shRNA models have been especially advantageous, allowing us to identify some of the genes that participate in the pathogenesis of Ewing tumors, such as cholecystokinin, DKK1 and the orphan nuclear receptor DAX1/NR0B1 [8], [9], [20].

EWS/FLI1 induced genes are expected to work functionally like oncogenes, while EWS/FLI1 repressed genes are expected to act functionally like tumor supressor genes. It is interesting that although EWS/FLI1 was shown to act as a potent transcriptional activator [21], [22], a significant proportion of EWS/FLI1 target genes are downregulated by this oncogenic protein [11], [23], [24]. The mechanism of this specific gene repression is only partially understood, and probably involves direct repression [11], [23]–[25], upregulation of transcriptional repressors [26] and epigenetic mechanisms [15]. In addition, EWS/FLI1 has been also shown to regulate the expression of microRNAs that in turn are available to regulate the expression of other genes involved Ewing sarcoma tumorigenesis [27], [28].

Analysis of our gene expression profile dataset in the Ewing sarcoma cell line A673 upon EWS/FLI1 knockdown showed that one of the most strongly downregulated genes by EWS/FLI1 codes for the enzyme lysyl oxidase (LOX). LOX is the major member of a family of lysyl oxidases (that include LOX and the LOX-like proteins LOXL1 to LOXL4) that share the enzyme catalytic domain (reviewed in [29]–[32]). LOX is synthesized as a 50-KDa inactive pre-proenzyme which is secreted into the extracellular environment and then processed by proteolytic cleavage to a functional 32-KDa LOX enzyme (LOXenz) and an 18-KDa propeptide (LOX-PP). It is well characterized that the functional mature enzyme catalyses lysine-derived covalent cross-links required for normal structural integrity of the extracellular matrix and a huge amount of information on this function, both in physiological and pathological situations, has been accumulated during several decades (reviewed in [29]–[32]). The role of the propeptide has been, however, much less studied, although recent reports suggest that LOX propeptide acts as a tumor suppressor in several contexts [33]–[35]. Currently, no data are available about the possible contribution of LOX repression to the malignant phenotype of Ewing sarcoma.

In this work, we show that EWS/FLI1 downregulates LOX expression and that, remarkably, LOX propeptide exhibits tumor suppressor activities in Ewing tumor cells. Ectopic expression of LOX propeptide in an Ewing sarcoma cell line reduced cell proliferation, cell migration, anchorage independent growth and tumor growth *in vivo*. These findings suggest that therapeutic strategies based in the administration of LOX propeptide or functional analogues could be useful for the treatment of this devastating paediatric cancer.

## Results

During the last years we have used an inducible model of EWS/FLI1 knockdown in combination with whole gene expression analysis to identify and characterize EWS/FLI1 target genes relevant for Ewing sarcoma tumorigenesis ([8], [9], [36]). Review of these datasets (GEO accession number GSE36007) indicated that one of the genes that showed a more intense and consistent downregulation by EWS/FLI1 was the enzyme lysyl oxidase (LOX, Protein-lysine 6-oxidase EC 1.4.3.13). Consequently, we decided to study the regulation of LOX by EWS/FLI1 and the functional implications of this downregulation.

**Figure 1:**
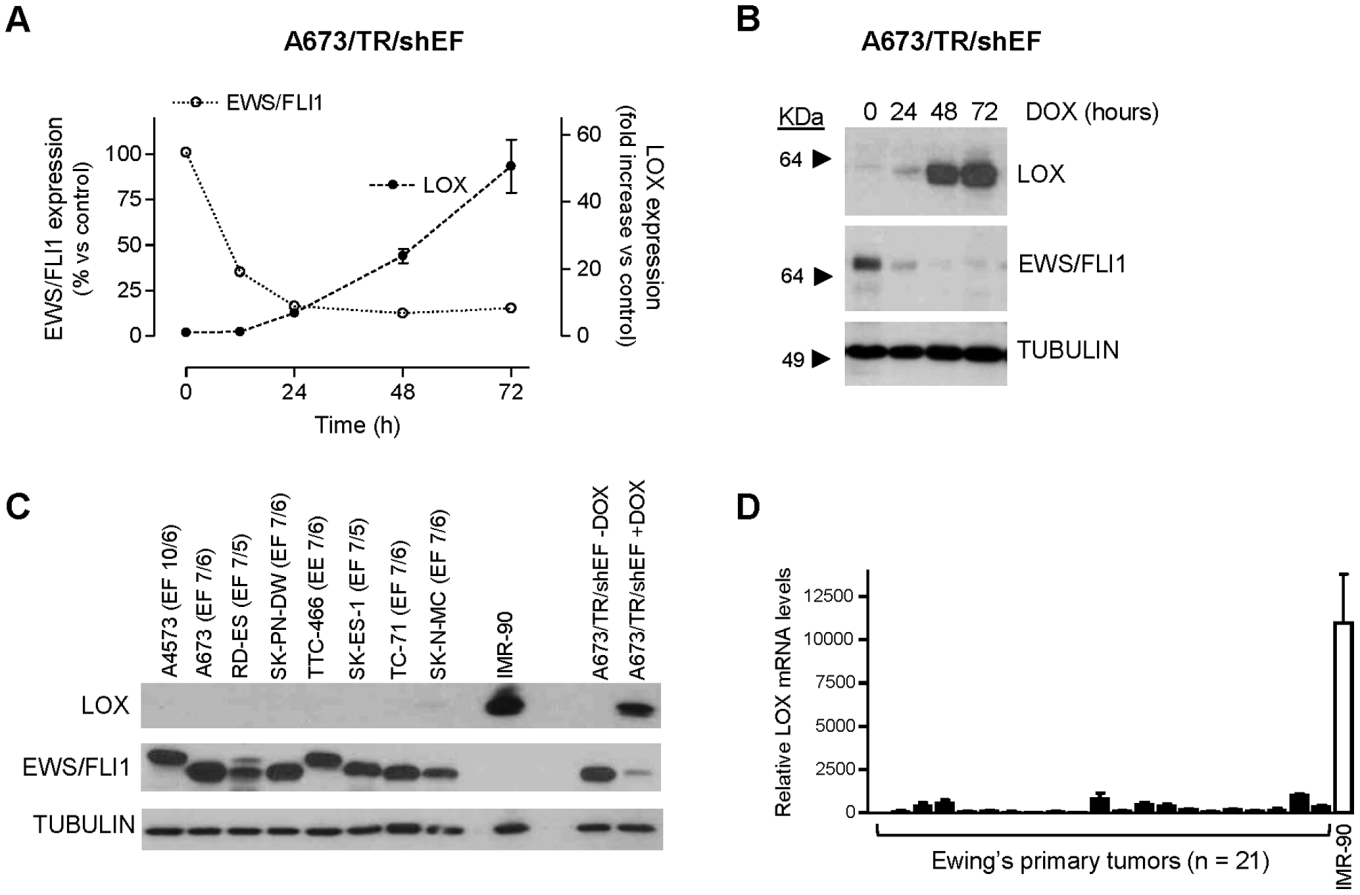
LOX mRNA and protein are downregulated by EWS/FLI1 in the A673 Ewing sarcoma cell line. **A**) A673/TR/shEF cells were stimulated by different periods of time with doxycycline (DOX, 1 µg/ml) to induce the expression of an EWS/FLI1-specific shRNA. The levels of LOX, EWS/FLI1 and TBP (reference gene) mRNA were quantified by multiplex real time qRT-PCR. For each time point, LOX and EWS/FLI1 mRNA levels were normalized to that of TBP and referred to unstimulated cells. The figure shows the data (mean±SD) of one out of two independent experiments done in triplicate with equivalent results. **B**) LOX protein levels were also determined by western-blot in the A673/TR/shEF cells stimulated with doxycycline. The same blot was stripped and successively incubated with anti-FLI1 antibody to assess the expression of EWS/FLI1 and with anti-α-tubulin as a control for loading and transferring. LOX mRNA and protein levels were strongly downregulated by EWS/FLI1. **C**) LOX protein levels were determined by western-blot in 8 Ewing derived cell lines and in normal fibroblasts IMR-90, used here as a positive control of LOX expression. A673/TR/shEF cells stimulated with doxycycline for 48 hours were also included. The type of EWS/FLI1 (EF; EWS exon/FLI1 exon) and EWS/ERG fusion (EE; EWS exon/ERG exon) present in each Ewing cell line are indicated. LOX protein expression was nearly undetectable in Ewing derived cell lines. **D**) LOX mRNA levels were quantified by qRT-PCR in a set of Ewing's primary tumors (n = 21). Normal fibroblasts IMR-90 are used as a positive control. mRNA levels were normalized to that of TBP (mean±SD). LOX mRNA levels observed in Ewing tumors were very low compared to those observed in the control fibroblast cell line IMR-90.

To confirm microarray data, we used A673/TR/shEF Ewing cells, which in response to doxycycline express a specific shRNA directed against EWS/FLI1 mRNA, subsequently reducing the levels of EWS/FLI1 mRNA and protein [8], [9]. We isolated RNA from A673/TR/shEF cells incubated in absence or in presence of doxycycline and performed real time quantitative RT-PCR (qRT-PCR) analysis. Time-course experiments confirmed that LOX is a gene downregulated by EWS/FLI1, since LOX mRNA levels were significantly upregulated upon EWS/FLI1 knockdown in the A673 Ewing sarcoma cell line (Figure 1A). As shown in figure 1B, LOX protein was nearly undetectable in A673/TR/shEF cells in basal conditions. However, EWS/FLI1 knockdown produced a dramatic increase in LOX protein levels confirming that EWS/FLI1 downregulates LOX expression in these cells. To analyse if LOX expression downregulation is a common feature of Ewing cells, we analysed by western-blot the levels of LOX protein in a panel of Ewing derived cell lines (n = 8) harbouring different oncogenic fusion proteins. As shown in Figure 1C, LOX expression in these Ewing sarcoma cell lines was nearly undetectable, while high LOX expression was observed in IMR-90 normal fibroblasts, which were used here as a positive control of LOX expression. We then analysed the levels of LOX mRNA in Ewing primary tumors using qRT-PCR and compared them with the levels observed in the positive control IMR-90. Figure 1D shows that LOX mRNA levels are low in Ewing primary tumors compared to those observed in IMR90 cells. In addition, we searched public expression datasets in order to analyse the relative expression of LOX in Ewing sarcoma in relation to other tumor types. As shown in supplementary figure S1, LOX expression was low in Ewing sarcoma tumors, compared to other neoplasms.

**Figure 2:**
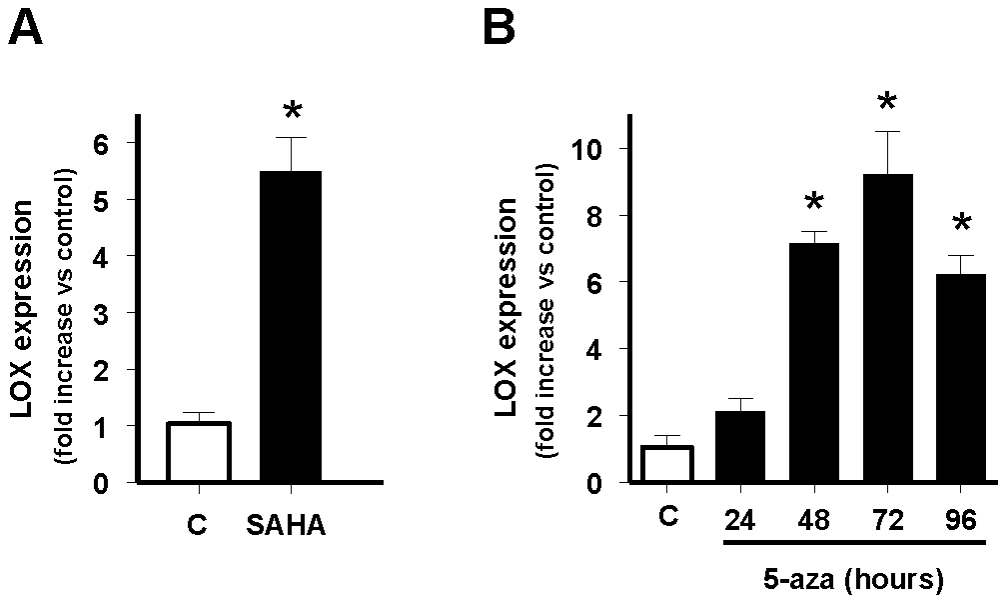
Effect of the histone deacetylases inhibitor SAHA and the demethylating agent 5-aza-2′-deoxycytidine on LOX mRNA levels. A) A673 cells were incubated in absence (C) or presence of SAHA 1 µM for 24 hours and the levels of LOX and TBP (reference gene) mRNAs quantified by multiplex real time qRT-PCR. LOX mRNA levels were normalized to that of TBP and referred to unstimulated control (C) cells, which were arbitrary set to 1. The figure shows the data (mean±SD) of three independent experiments done in triplicate. The figure shows the mean±SD of one out of two independent experiments done in duplicate (*P<0.01 vs control). B) A673 cells were incubated in the absence (C) or presence of 5-aza-2**′**-deoxycytidine 1 µM for 24, 48, 72 or 96 hours and the levels of LOX and TBP (reference gene) mRNAs quantified by qRT-PCR and analysed as above. The figure shows the mean±SD of two independent experiments done in duplicate (*P<0.01 vs control). Both SAHA and 5-aza-2**′**-deoxycytidine produce a significant increase in the levels of LOX mRNA in A673 cells.

Next, we analysed if epigenetic mechanisms, known to repress gene expression, could be involved in the negative regulation of LOX expression observed in Ewing sarcoma cells. We first studied the effect of Vorinostat (SAHA), a class I/II histone deacetylase inhibitor approved for clinical use in cancer patients ([37]). A673 cells were incubated in presence or absence of SAHA for 24 hours and LOX mRNA levels were quantified by qRT-PCR. As shown in Figure 2A, incubation of A673 Ewing cells with SAHA produced a 5-fold increase in LOX mRNA levels. Following, we analysed the effect of 5-aza-cytidine (5-aza), a potent inhibitor of DNA methyltransferase 1 (DNMT1) that induces demethylation and reactivation of silenced genes [38] on LOX expression. As shown in figure 2B, incubation of A673 Ewing cells with 5-aza during 72 hours produced a 10-fold increase in LOX mRNA levels. These results suggest that both histone acetylation status and DNA methylation could be involved in the negative regulation of LOX expression in A673 cells.

The fact that EWS/FLI1 downregulates LOX in the A673 Ewing sarcoma cell line and that low levels of LOX are a common feature of Ewing sarcoma cells and tumors, suggest that LOX could act as a tumor suppressor in Ewing tumors. In this case, LOX re-expression should antagonize, at least in part, the transforming properties of EWS/FLI1 in Ewing tumor cells. To confirm this hypothesis, we performed functional studies to determine the effect of LOX re-expression in the A673 Ewing cell line model.

**Figure 3:**
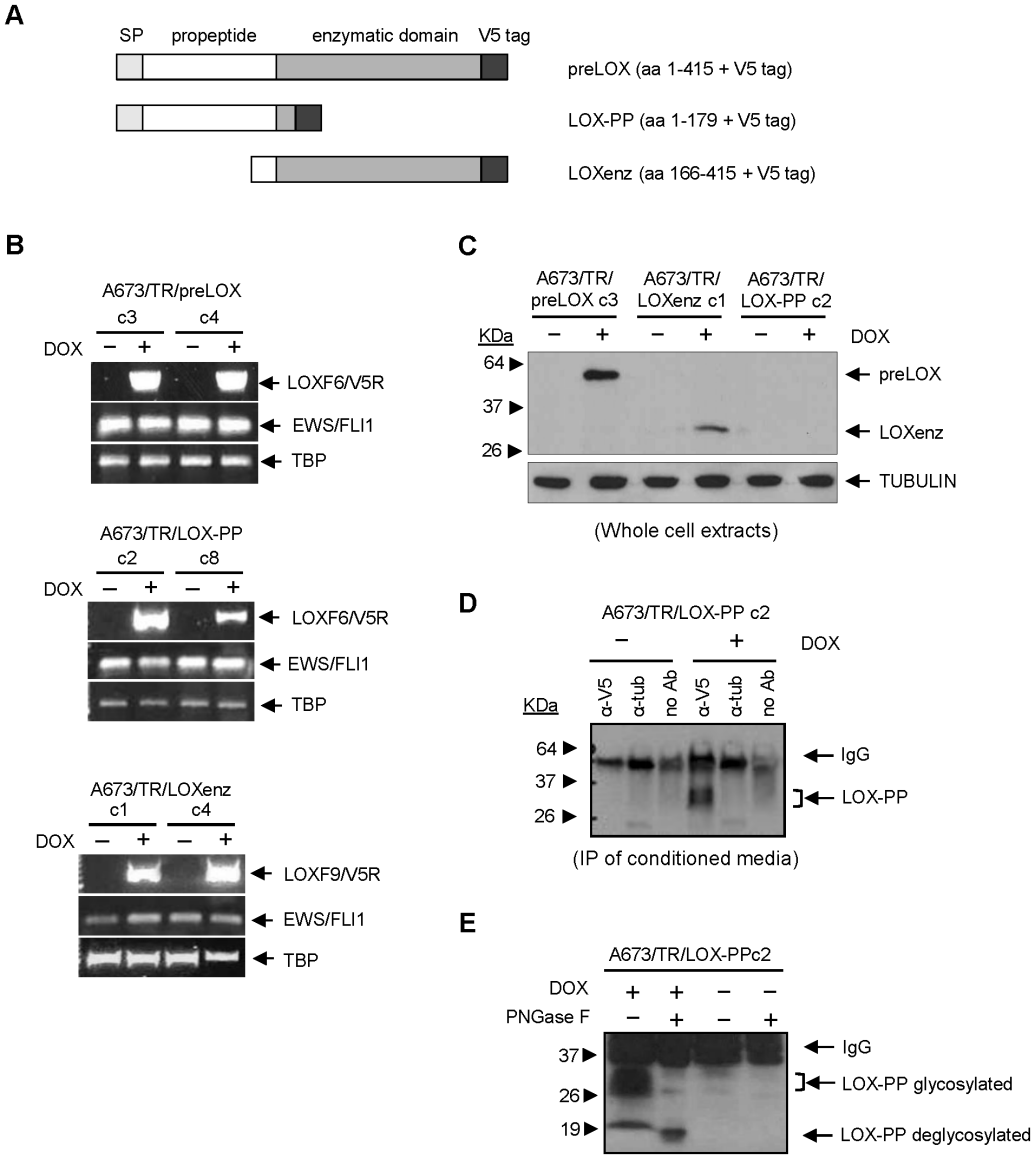
Establishment of Ewing sarcoma cell lines expressing doxycycline-inducible preLOX, LOX-PP and LOXenz proteins. **A**) Schematic representation of preLOX, LOX-PP and LOXenz cDNAs cloned in the doxycycline-inducible lentiviral vector pLenti-TO-V5-DEST. SP: signal peptide. **B**) A673/TR Ewing cell line expressing high levels of the tetracycline repressor (TR) was infected with the doxycycline-inducible lentiviral vector encoding the different LOX cDNAs and stable clones were selected. The figure shows two independent clones for each construction stimulated with doxycycline (DOX, 1 µg/ml, 48 hours) to induce the expression of the corresponding mRNAs. preLOX, LOX-PP and LOXenz mRNAs were detected by RT-PCR using LOX-specific (LOXF6 or LOXF9) and V5 tag-specific primers (V5R). EWS/FLI1 fusion mRNA remained unchanged upon LOX induction. TBP (TATA-binding protein) was used as an internal housekeeping control. **C**) Whole protein extracts were isolated from A673/TR/preLOX (clone 3), A673/TR/LOXenz (clone 1) and A673/TR/LOX-PP (clon 2) cells stimulated with doxycycline (DOX, 1 µg/ml, 48 hours) and analysed by western-blot to detect the expression of preLOX, LOXenz and LOX-PP proteins, respectively, using an anti-V5 antibody. The same blot was stripped and incubated with anti-α-tubulin as a control for loading and transferring. preLOX and LOXenz, but not LOX-PP, were detected in whole cell extracts. **D**) Conditioned media derived from A673/TR/LOX-PP (clone 2) incubated with doxycycline (DOX, 1 µg/ml, 48 hours) was concentrated and immunoprecipitated with an anti-V5 antibody to confirm the secretion of LOX-PP in the culture media. An anti-α-tubulin or no antibody were used as negative controls. LOX-PP was detected in the culture media derived from A673/TR/LOX-PP stimulated with doxycycline indicating that LOX-PP was efficiently processed and secreted. **E**) Conditioned media derived from A673/TR/LOX-PP cells stimulated with doxycycline was immunoprecipitated and untreated or treated with the enzyme peptide-N-glycosidase (PNGase F). Glycosylated LOX-PP was observed as a diffused band of approximately 30 KDa in the untreated sample, that became a defined band of 18 KDa upon deglycosylation treatment. A673/TR/LOX-PP cells without doxycycline were used as a negative control of LOX-PP production.

As previously mentioned, LOX is synthesized as a 50-KDa inactive proenzyme (preLOX) which is secreted to the extracellular environment. There, it is processed by proteolytic cleavage to a functional 32-KDa LOX enzyme (LOXenz) and an 18-KDa propeptide (LOX-PP). We thus genetically modified the A673 Ewing sarcoma cell line to express LOX proenzyme (A673/TR/preLOX, aminoacides 1-415), its catalytic domain (A673/TR/LOXenz, aminoacides 166-415) and the LOX propeptide (A673/TR/LOX-PP, aminoacides 1-179) in an attempt to characterize the specific contribution of full-length LOX and of each one of the LOX-derived fragments to Ewing sarcoma tumorigenesis (Figure 3A). We have used a doxycycline-inducible system to express these proteins because, in our opinion, it has significant advantages to analyse genes that may be acting as tumor suppressors since constitutive expression of these genes is expected to produce deleterious effects on transformed cells.

As shown in Figure 3B, preLOX, LOXenz and LOX-PP mRNAs were induced upon doxycycline stimulation in the A673/TR/preLOX, A673/TR/LOXenz and A673/TR/LOX-PP Ewing cells respectively. In all cases, levels of EWS/FLI1 mRNA were not altered by doxycycline stimulation, making this an excellent model to analyse the effect of the induction of the different LOX proteins and their contribution to Ewing pathogenesis. Western-blot analyses were used to confirm the expression of the corresponding proteins: preLOX and LOXenz were detected by western-blot in whole cell extracts as proteins with molecular weights slightly larger than expected, because of the V5 N-terminal tag sequence that was fused to the proteins to facilitate their detection with an anti-V5 specific antibody (Figure 3C). In contrast, LOX-PP was not detected in whole cell extracts (Figure 3C), suggesting that it was rapidly and efficiently secreted to the culture media. To confirm this, we used immunoprecipitation to analyse the culture media of A673/TR/LOX-PP cells incubated in absence or in presence of doxycycline. As shown in figure 3D, LOX-PP was detected as a diffuse band of approximately 30 KDa in culture media of A673/TR/LOX-PP cells stimulated with doxycycline, suggesting that this protein was strongly glycosylated, as previously reported [39]–[41]. In order to demonstrate that this diffuse band corresponds to secreted LOX-PP highly glycosylated, we treated a fraction of the immunoprecipitated LOX-PP with the enzyme N-glycosidase (PNGase F) to remove N-linked oligosaccharides. After N-glycosidase treatment (Figure 3E), LOX-PP migrated as a single band of approximately 18 kDa, which corresponds to its predicted size according to its amino acid composition and demonstrates that LOX-PP produced by A673/TR/LOX-PP cells is processed adequately. Unglycosylated LOX-PP was also observed in untreated LOX-PP, although it was present in a very low quantity, indicating that a minimal fraction of the secreted LOX-PP was unglycosylated.

**Figure 4:**
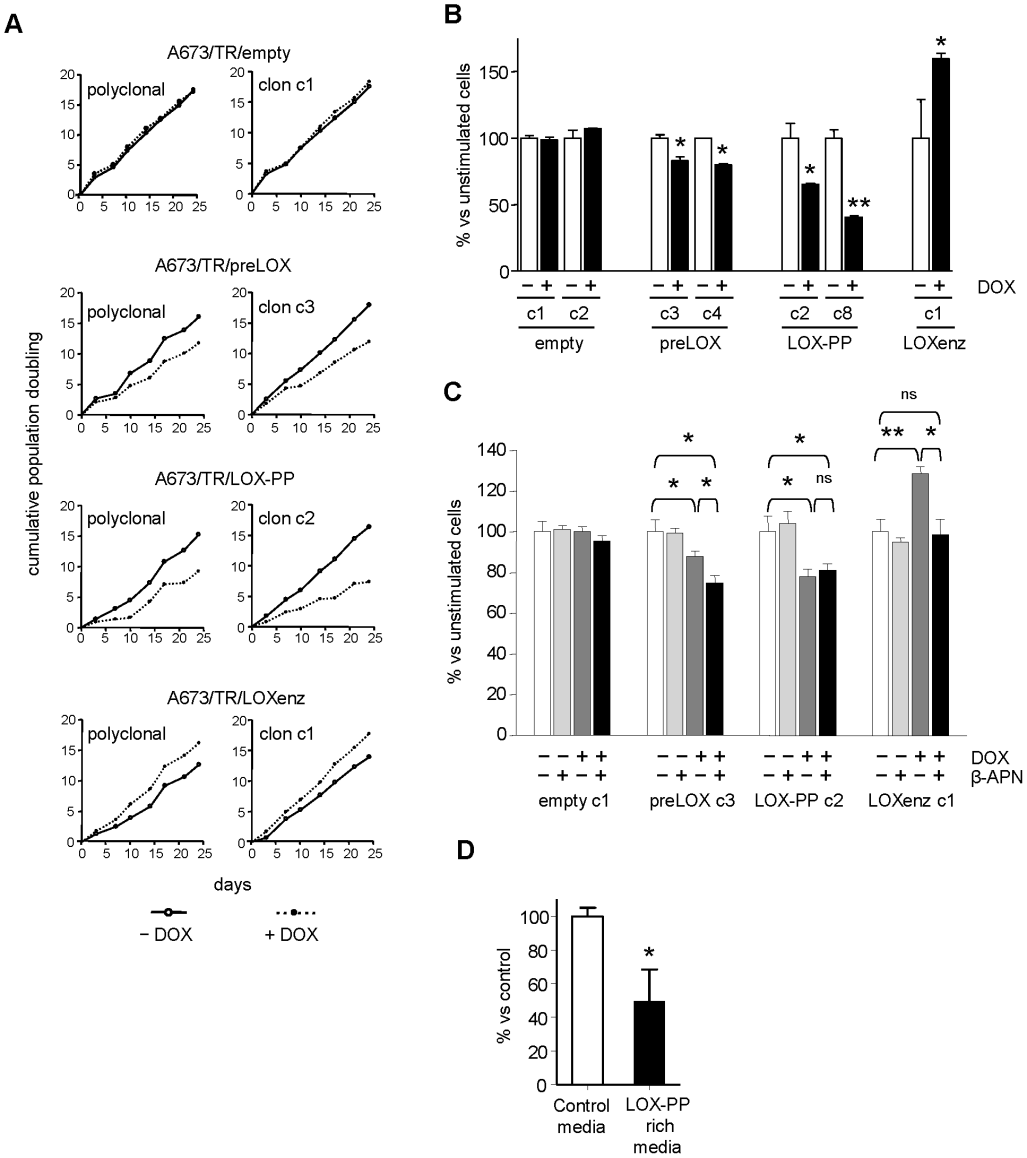
Induction of preLOX and LOX-PP inhibits cell proliferation in A673 Ewing sarcoma cells. **A**) A polyclonal population and a representative clone of A673/TR/empty (control cells), A673/TR/preLOX, A673/TR/LOX-PP and A673/TR/LOXenz cells were maintained during 25 days in standard culture medium or in culture medium containing doxycycline (DOX, 1 µg/ml) to induce the expression of the corresponding proteins. Cumulative number of population doubling were determined by counting cells at different periods of time and plotted versus time. **B**) Representative clones of A673/TR/empty, A673/TR/preLOX, A673/TR/LOX-PP and A673/TR/LOXenz cells were incubated in the absence or in the presence of doxycycline (DOX, 1 µg/ml) for 5 days to induce the corresponding proteins and cell growth quantified by CellTiter-Fluor assay. The figure shows the mean±SD of one out of three independent experiments done in triplicate with similar results. Data are shown as percentage versus unstimulated cells, which were arbitrarily set to 100 (*P<0.05, **P<0.005 versus unstimulated cells). **C**) A673/TR/empty, A673/TR/preLOX, A673/TR/LOX-PP and A673/TR/LOXenz cells were incubated in the absence or in the presence of doxycycline (DOX, 1 µg/ml) and in the absence or in the presence of the irreversible antagonist of lysyl oxidase activity β-aminopropionitrile (β-APN, 500 µM). The figure shows the mean±SD of one out of two independent experiments done in triplicate with equivalent results. Data are shown as percentage versus unstimulated cells, which were arbitrarily set to 100 (*P<0.05, **P<0.005, ns: not significant). **D**) A673 cells were incubated during 72 hours with conditioned media derived from A673/TR/LOX-PP cells cultured in absence of doxycycline (control) or conditioned media derived from the same cells cultured in presence of doxycycline to induce the expression of LOX-PP (LOX-PP rich media). The figure shows the mean±SD of two independent experiments done in triplicate. Data are shown as percentage versus cells incubated with control media, which were arbitrarily set to 100 (*P<0.05). LOX-PP rich media significantly reduced cell proliferation of A673 cells.

We next analyzed the effect of preLOX, LOX-PP and LOXenz on cell proliferation. As shown in Figure 4, induction of preLOX by doxycycline in A673/TR/preLOX cells resulted in a significant reduction (30%) in the number of population doubling accumulated during 25 days. This inhibitory effect on cell proliferation was clearly more pronounced (45%) when LOX-PP was induced in A673/TR/LOX-PP cells. Interestingly, the effect of LOXenz on cell proliferation was the opposite, with an increase in the number of population doubling upon LOXenz induction. No effect on cell proliferation was observed in A673 cells carrying the empty vector, both in absence and in presence of doxycycline. To confirm these effects on cell proliferation, we performed additional experiments using an available commercial kit designed to quantify viable cells (Cell-titer fluor cell viability assay). As shown in Figure 4B, induction of preLOX in two independent clones of A673/TR/preLOX cells produced a reduction (about 20%) in the number of viable cells. This inhibitory effect on cell number became again more pronounced (about 50%) when LOX-PP was induced in A673/TR/LOX-PP cells. By contrast, the induction of LOXenz resulted in an increase in the number of viable cells. Taken together, these results demonstrate that preLOX inhibits cell proliferation in Ewing cell lines and more interestingly, that this inhibitory effect on cell proliferation resides in the LOX propeptide. To additionally characterize these findings, we used the irreversible antagonist of lysyl oxidase activity β-amino-propionitrile (β-APN) [33]. As shown in Figure 4C, treatment with β-APN increased the inhibitory effect of preLOX on cell proliferation. Since preLOX expression results in the production of both the propeptide domain LOX-PP and the catalytic domain LOXenz, this result indicates that lysyl oxidase activity (which resides in LOXenz) is partially counteracting the cell proliferation inhibition mediated by LOX-PP. Furthermore, β-APN totally blocked the stimulatory effect of LOXenz on cell proliferation, demonstrating that this effect is entirely dependent on the lysyl oxidase catalytic activity. In concordance with this, β-APN had no effect on the cell proliferation inhibition observed upon LOX-PP induction. These results confirm that the inhibitory effect of LOX on cell proliferation resides in the LOX propeptide. Subsequently, we analyzed the effect of an exogenous source of LOX-PP on cell proliferation. For this purpose, we incubated A673 cells with LOX-PP rich media, obtained from conditioned media of A673/TR/LOX-PP cells stimulated with doxycycline during 72 hours. Control media, without LOX-PP, was obtained in parallel from the same cells incubated in absence of doxycycline for 72 hours. As shown in figure 4D, LOX-PP enriched media, reduced by 50% the cell number in A673 Ewing cells.

**Figure 5:**
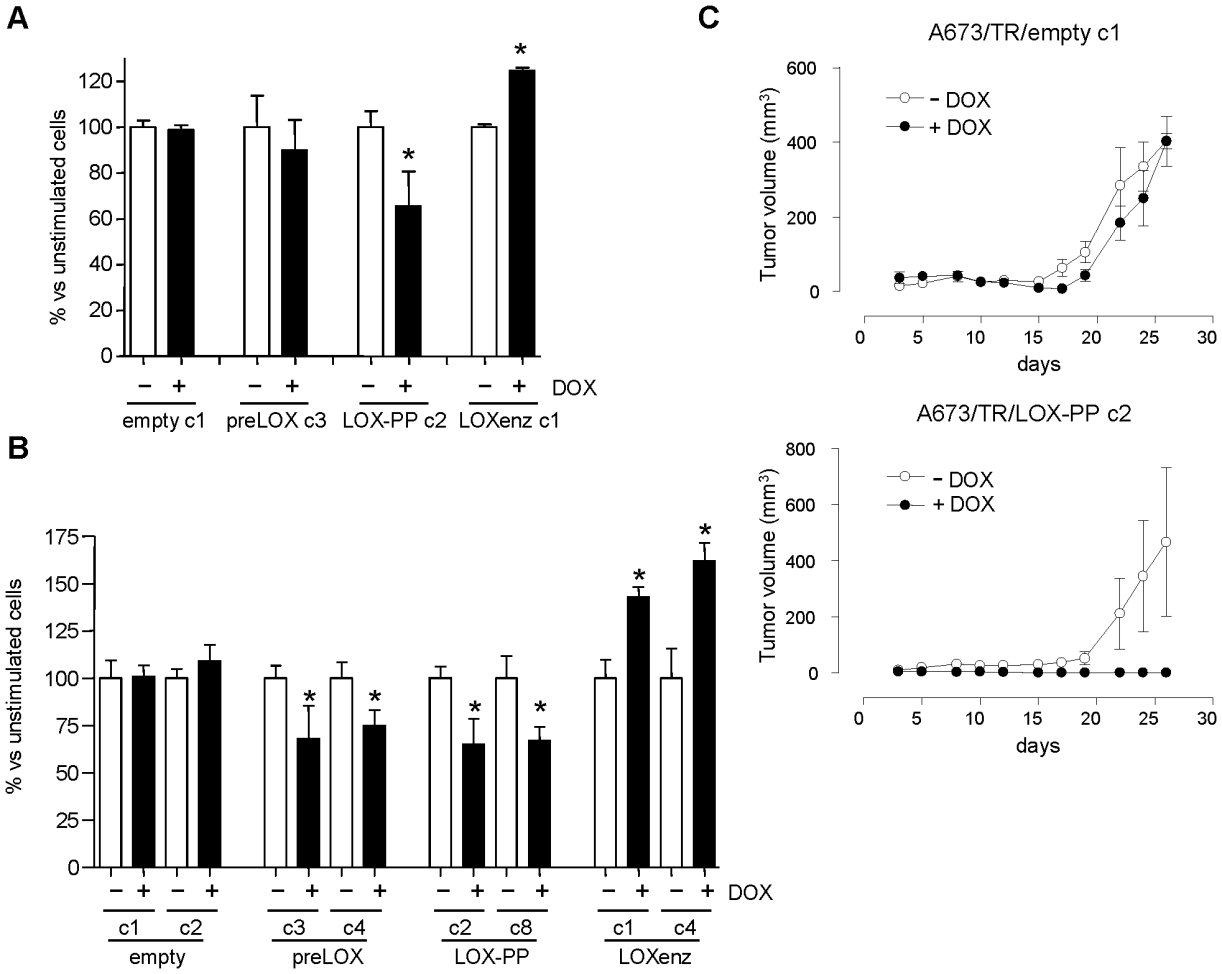
LOX-PP induction inhibits growth in soft agar, cell migration and tumor growth *in vivo*. **A**) A673/TR/empty, A673/TR/preLOX, A673/TR/LOX-PP and A673/TR/LOXenz cells were grown in soft agar in the absence or in the presence of doxycycline (DOX, 1 µg/ml)) for 25 days. Culture dishes were then photographed and colony number was calculated. The figure shows the mean±SEM of three independent experiments done in triplicate. Data are shown as percentage versus unstimulated cells, which were arbitrarily set to 100 (*P<0.05, versus unstimulated cells). **B**) Two representative clones of A673/TR/empty, A673/TR/preLOX, A673/TR/LOX-PP and A673/TR/LOXenz cells were incubated in the absence or in the presence of doxycycline (DOX, 1 µg/ml) during 48 hours to induce the expression of the corresponding proteins. Afterwards, cells were placed in the upper compartment of a transwell and allowed to migrate through the membrane in response to serum. Migrating cells were quantified by crystal violet staining. The figure shows the mean±SEM of one experiment done in triplicate. Data are shown as percentage versus unstimulated cells, which were arbitrarily set to 100 (*P<0.05, versus unstimulated cells). **C**) Nude mice were injected with A673/TR/empty clone 1 (n = 7) or A673/TR/LOX-PP clone 2 (n = 8) cells and split in two groups, one of which was given doxycycline (DOX, 1 mg/ml) in the drinking water to induce the expression of the corresponding protein. The figure shows the evolution of tumor volume (mean±SEM of 3–4 animals per group) versus time.

Following, we analysed the effect of preLOX, LOX-PP and LOXenz on cell migration and the ability to grow in an anchorage-independent manner. In Figure 5A are shown the effects of preLOX, LOX-PP and LOXenz on cell migration through porous membranes in response to serum. Induction of preLOX with doxycycline in the A673/TR/preLOX cells did not produce a significant difference in the number of cells migrating through the membrane. However, the induction of LOX-PP in A673/TR/LOX-PP cells decreased cell migration through the porous membranes by 35%, indicating that LOX-PP is also active in inhibiting cell migration. In this case, LOXenz also showed the opposite effect when compared to LOX-PP. Thus, LOXenz increased the migration of A673 cells by 25%. Afterwards, we analysed the effect of preLOX, LOX-PP and LOXenz on the ability of A673 cells to grow in soft agar. As shown in Figure 5B, preLOX and LOX-PP induction decreased the number of colonies grown in soft agar by approximately 25% and 30%, respectively. By contrast, LOXenz increased the number of colonies by about 50%.

Subsequently, we performed xenograft experiments to analyse the effect of LOX-PP on tumor growth *in vivo* (Figure 5C). Mice were injected subcutaneously with A673/TR/empty or A673/TR/LOX-PP and each group of animals was split into two groups of treatment, one of which was given doxycycline in the drinking water to induce the expression of the corresponding protein. As expected, animals injected with A673/TR/empty cells produced tumors both in the absence and in the presence of doxycycline in the drinking water. Animals injected with A673/TR/LOX-PP cells but not treated with doxycycline also developed tumors at the same rate as the animals injected with the A673/TR/empty control cells. However, we did not detect visible tumors in the animals injected with A673/TR/LOX-PP cells and treated with doxycycline, and thus expressing LOX-PP. Taken all together these results indicate that LOX-PP is a suppressor of Ewing sarcoma tumorigenesis.

**Figure 6:**
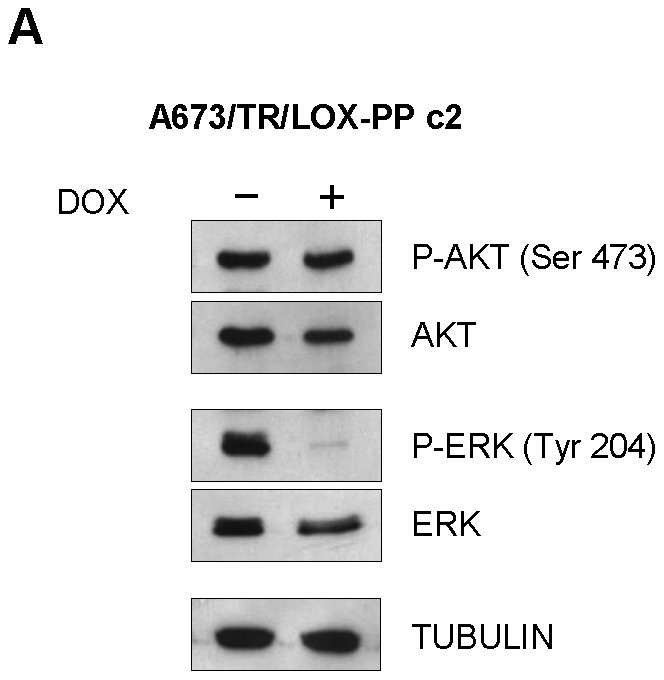
LOX-PP interferes with ERK signalling. A673/TR/LOX-PP cells (clone 2) were incubated in the absence or in the presence of doxycycline (DOX, 1 µg/ml, 48 hours) and the levels of activates P-AKT (Ser 473) and P-ERK (Tyr 204) determined by western-blot. The same blot was stripped and successively incubated with anti-AKT, anti-ERK and anti-α-tubulin as controls for loading and transferring.

Following, in an attempt to identify the pathways involved in the antiproliferative effect of LOX-PP we analysed by western-blot the status of the PI3K/AKT and ERK/MAPK pathways. As shown in Figure 6, induction of LOX-PP with doxycycline had no effect on the levels of activated Akt (P-Ser 473). By contrast, LOX-PP produced a marked inhibition of the levels of activated Erk (P-Tyr 204).

Finally, we performed a gene expression microarray experiment to identify genes and pathways specifically regulated by LOX-PP in A673 cells (GEO accession number GSE46407). Thus, we stimulated A673/TR/LOX-PP cells with doxycycline for 72 hours and compared its gene expression profile to that of the control cells A673/TR/empty. In order to identify pathways specifically affected by LOX-PP in A673 cells, we analysed the expression data sets with GSEA (Gene Set Enrichment Analysis) [42]. As shown in supplementary table S1, GSEA analysis identified many gene sets/pathways from the REACTOME curated database (www.reactome.org) that were significantly regulated by LOX-PP (FDR<0.005). Among the 19 gene sets displaying a FDR<0.005, 14 were related to DNA synthesis and replication and cell cycle regulation (Supplementary table S1 and figure S2), indicating that LOX-PP was clearly affecting cell proliferation. LOX-PP induction also affected other pathways related to cell metabolism (3 gene sets) and extracellular matrix organization (2 gene sets). Taken together, these data suggest that LOX-PP is affecting cell proliferation, first, by producing a significant inhibition of the ERK/MAPK pathway, and subsequently, by affecting pathways involved in cell cycle progression.

## Discussion

Ewing sarcoma is a highly aggressive paediatric cancer that in an elevated percentage of cases is refractory to standard treatments (reviewed in [1]). Therefore, studies designed to identify new therapeutic targets in Ewing tumors are eagerly needed. Ewing sarcoma is characterized by chromosomal translocations that lead to the expression of chimeric transcription factors (the most frequent of which is the fusion protein EWS/FLI1), which are thought to be the responsible for tumor initiation [3]–[5]. Because EWS/FLI1 (and the related fusion proteins) deregulates a large set of target genes, identification of these genes is not only important to increase our understanding about Ewing tumorigenesis, but more importantly to identify new targets that could be pharmacologically modulated.

Whole genome expression analysis has been especially useful to identify EWS/FLI1 target genes and to provide a relevant amount of information about genes and pathways involved in Ewing tumorigenesis [43]. However, in order to identify new valuable therapeutic targets, functional analysis should be performed “gene to gene” to establish the individual contribution of each gene to the malignant phenotype. With this in mind, in this work we analysed the contribution of lysyl oxidase (LOX), a EWS/FLI1 downregulated gene target, to Ewing tumorigenesis.

Microarray studies and real time quantitative RT-PCR demonstrated that LOX mRNA was downregulated by the EWS/FLI1 oncoprotein in the A673 Ewing sarcoma cell line. Our results are consistent with other previously reported data, in which EWS/FLI1 was also shown to downregulate LOX expression in A673 and other Ewing cell lines (i.e. EWS502 and TC-71) [11], [17], [18], [44]. In agreement with this, we observed no expression (or nearly undetectable) of LOX protein in eight different Ewing cell lines. In addition, LOX mRNA levels were also low in a series of Ewing primary tumors, which was also confirmed searching public available datasets. Loss of LOX expression has been reported in a variety of human cancers. More than two decades ago, Kuivaniemi et al. [45] observed deficient LOX production in a variety of malignant transformed cells, such as HT-1080 (fibrosarcoma), A-204 (rhabdomyosarcoma) or G-361 (melanoma). More recently, reduced LOX expression has been reported to occur in lung and pancreatic cancer [46], [47] and during malignant progression of prostate and colorectal cancers [48], [49]. Therefore, at least in some types of tumors, LOX expression correlates negatively with malignant transformation.

Our results suggest that epigenetic mechanisms could be involved in the downregulation of LOX mRNA in A673 Ewing cells. In this sense, both the histone deacetylase inhibitor SAHA and the demethylating agent 5-aza-citidine restored, at least partially, LOX expression in A673 cells. It is difficult to assess the quantitative contribution of these mechanisms to LOX repression, because of the elevated toxicity of these agents, in particular SAHA, but our results indicate that both could be involved. While we were doing this work, Lessnick's lab described that EWS/FLI1 oncoprotein was directly involved in the repression of LOX promoter [25]. Interestingly, the repression of LOX promoter was dependent on the interaction of EWS/FLI1 with the NuRD repressor complex. In concordance with this, Sankar et al, showed that LOX mRNA levels increased upon incubation of A673 cells with SAHA, in agreement with our results. In addition, we also showed that DNA methylation can be involved in the repression of LOX gene in A673 cells, although we did not demonstrate if this was an indirect or a direct mechanism.

One of the most interesting findings of our work is the demonstration that lysyl oxidase and particularly, the lysyl oxidase propeptide (LOX-PP) is a suppressor of Ewing sarcoma tumorigenesis. Expression of LOX propeptide decreased cell proliferation, migration, colony formation in soft agar and growth of tumors *in vivo*. In addition, conditioned media containing high levels of LOX-PP impaired cell proliferation in A673 Ewing cells. By contrast, expression of the LOX domain containing the lysyl oxidase activity (namely here LOXenz) showed the opposite effects. Interestingly, when full-length LOX protein (preLOX) was expressed in Ewing tumor cells, the inhibitory effect of LOX-PP on cell proliferation was dominant on the stimulatory effect mediated by LOXenz. Thus, preLOX expression produced a net inhibitory effect on cell proliferation, which could explain why LOX downregulation is advantageous for Ewing tumor cells. Taken together, our results demonstrate that LOX propeptide is a suppressor of Ewing tumorigenesis. Although we have performed the experiments with the prototype Ewing cell line A673, we anticipate that LOX-PP will also affect the growth and transforming properties of other Ewing cell lines. In this sense, Sankar et al have shown in their recently published article that LOX (preLOX) overexpression reduced the ability of three Ewing cell lines (A673, TC71, TC32) to form colonies in soft agar [25]. Although in that study it was not analysed the effect of LOX propeptide itself, these results, taken together with ours, suggest that LOX-PP could act as an antitumor agent for the majority of Ewing sarcoma cells.

The first evidence about the tumor suppressor activity of LOX came from studies addressed to identify the genes involved in the IFN-γ mediated-reversion of ras-transformed malignant cells. In these studies, a gene called ras recision gene (rrg) was isolated and shown to be responsible for malignant reversion [50]. In agreement with this, increased LOX expression was also found in spontaneous revertants of H-ras transformed rat fibroblasts [51] while reduced LOX expression was observed in ras-transformed cells [52]. In addition, Giampuzzi et al. reported that normal rat kidney fibroblasts (NRK-49F) in which LOX mRNA was knocked down by antisense lysyl oxidase showed loose attachment to the plate and anchorage-independent growth and were highly tumorigenic in nude mice [53]. These findings evidence a role of LOX as a tumor suppressor, highlighting its particular role in controlling Ras activation and growth factor dependence.

Palamakumbura et al, described for the first time that the lysyl oxidase propeptide, but not the lysyl oxidase enzyme, was responsible for the inhibition of ras-dependent transformation of NIH3T3 cells as determined by effects on cell proliferation assays, growth in soft agar and Akt-dependent induction of NF-kappaB activity [33]. Subsequently, LOX-PP was shown to act as a tumor suppressor in several cancer cells. For example, LOX-PP reverts the invasive phenotype of breast cancer cells [34], inhibits the transformed phenotype of lung and pancreatic cancer cells [35], interferes with FAK activation in breast cancer cells [54] and inhibits prostate cancer cell growth by targeting FGF-2 cell binding and signalling [55].

The mechanism through LOX-PP act as a tumor suppressor begins now to be only partially understood. LOX-PP has been shown to impair both PI3K/Akt and ERK signalling pathways. Thus, ectopic pre-LOX and LOX-PP expression in H1299 lung cancer and PANC-1 pancreatic cancer cells inhibited growth in soft agar and migration and reduced activation of ERK and Akt, with LOX-PP showing substantially higher activity [35]. In MIA PaCa-2 (a pancreatic cancer cell), LOX-PP attenuated the ERK and Akt activities and decreased the levels of the NF-Kβ p65 and RelB subunits and cyclin D1, which are activated by RAS signalling [56]. Palamakumbura et al. [55] showed that recombinant LOX-PP protein inhibits serum-stimulated DNA synthesis and ERK and PI3K/Akt pathways in DU 145 and PC-3 androgen-independent cell lines. Our data indicate that the inhibition of proliferation observed upon LOX-PP expression in A673 Ewing cells could be mainly mediated by inhibition of the ERK pathway, while the PI3K/Akt pathway does not seem to be involved. Therefore, specific downstream targets of LOX-PP can depend on the cellular context. Studies carried out with gene expression microarrays shown as well that LOX-PP modified the expression of genes involved in specific pathways related to DNA synthesis and replication and cell cycle regulation, indicating that LOX-PP was clearly affecting cell proliferation. These results suggest that LOX-PP is affecting cell proliferation, first, by producing a significant inhibition of the ERK/MAPK pathway, and subsequently, by affecting pathways involved in cell cycle progression.

In summary, in this work we shown that LOX is a gene repressed by the EWS/FLI1 oncoprotein and that its ectopic re-expression in an Ewing cell line inhibits cell proliferation. We also shown that this function is attributed to the LOX propeptide, an N-terminal fragment of the LOX protein released during processing, which in turn is able to decrease cell proliferation and migration and to inhibit the formation of colonies in soft agar and the growth of tumors *in vivo*. Our data indicates that LOX propeptide is a tumor suppressor gene in Ewing's tumors, providing the bases for a rational use of LOX propeptide, derived peptides or synthetic peptidomimetics in Ewing's therapy. Recently, it has been shown that LOX-PP sensitizes pancreatic and breast cancer cells to doxorubicin-induced apoptosis, providing a rationale for LOX-PP usage in adjuvant chemotherapy [56]. Thus, in light of the broad inhibitory activities of LOX-PP shown in this work, it will be interesting to further explore the use of LOX-PP as a potential treatment for Ewing tumors in combination with standard chemotherapy agents.

## Materials and Methods

### Ethics statement

Experiments with mice were all carried out in accordance with Institutional and European Union guidelines for the care and use of laboratory animals. Procedures were approved by the Comité de Ética de la Investigación y Bienestar Animal (CEIyBA) of the Instituto de Salud Carlos III (Certificate number: PA-24_2012). Animals were sacrificed using an overdose of sodium pentobarbital and all efforts were made to minimize suffering.

Tumors used in this study were provided by the Departments of Pathology and Oncology Units of several Spanish Children's Hospitals. A written informed consent was obtained from each patient's guardian. This study was approved by the Comité de Ética de la Investigación y Bienestar Animal (CEIyBA) of the Instituto de Salud Carlos III (Certificate number: PI- 14_2012).

### Cell lines

The Ewing sarcoma cell lines A673 (CRL-1598), SK-ES-1 (HTB-86), RD-ES (HTB-166), SK-N-MC (HTB-10), and SKPN-DW (CRL-2139) were purchased from the American Type Culture Collection (Manassas, VA, USA). Ewing sarcoma cell lines A4573 [57] and TTC-466 [58] were generous gifts from Dr. S. Navarro (University of Valencia, Valencia, Spain) and Dr. T.J. Triche (Children's Hospital Los Angeles, Los Angeles, CA), respectively.

### Materials

Stock solution of doxycycline (Invitrogen, Grand Island, NY, USA) were made in PBS 1x. Vorinostat (SAHA) (Sigma-Aldrich, St. Louis, MD, USA) was dissolved in DMSO to generate a stock solution and stored at −20°C until use. This solution was then dissolved in sterile PBS 1X to obtain the desired concentrations. 5-Aza-2**′**-deoxycytidine (Sigma-Aldrich) was dissolved in DMSO and stored at −80°C in small aliquots until use.

### Establishment of Ewing`s cell lines stably expressing doxycycline-inducible cDNA of LOX, LOX-PP and LOXenz

The complete sequence of LOX (LOX), the propeptide region of LOX (LOX-PP) and the mature form of LOX containing the enzymatic activity (LOXenz) were PCR-amplified from the LOX cDNA cloned into the vector pCMV6-XL5 (Origene, Rockville, USA). LOX cDNA (aminoacids 1-415) was amplified using primers LOX-F3 (5′-GGGGGATCCCAATC TGGCAAAAGGAGTGATGC-3**′**) and LOX-R3 (5**′**-GGGCTCGAGGAAATTGTGCAGCC TGAGGCATA-3**′** ); LOX-PP cDNA (aminoacids 1–179) was amplified using primers LOX-F3 and LOX-R7 (5**′**-GGGCTCGAGGTCAGAGTACTTGTAGGGGTTGTA-3**′** ); LOXenz cDNA (aminoacids 166–415) was amplified using primers LOX-F7 (5**′**-GGGGGATCCAGAAGTTCCTGCGCTCAGTAA-3**′** ) and LOX-R3. The amplified fragments were digested with BamHI and XhoI, cloned into the pENTR2B plasmid (Invitrogen) and transferred by recombination to the lentiviral doxycycline-inducible plasmid pLenti4-TO-V5-DEST (Invitrogen). Then, A673/TR Ewing sarcoma cells expressing the tetracycline repressor [8], [9] were infected with lentiviruses containing the corresponding cDNAs. Control cells were infected with empty lentiviral vector. Stable clones were selected with zeocin (100 µg/ml). Induction of LOX, LOX-PP and LOXenz were assayed by quantitative RT–PCR and/or western-blots upon doxycycline (1 µg/ml) stimulation. Clones displaying the highest levels of mRNA and/or protein expression upon doxycycline stimulation were chosen for additional studies.

### Multiplex real-time quantitative RT-PCR

Real-time PCR was performed to quantify steady state mRNA levels as described elsewhere [13]. Sequences of the primers and TaqMan probes used were as following: for EWS/FLI1, EWS/FLI1-F, 5**′**-AGCCAAGCTCCAAGTCAATATAG-3**′** , EWS/FLI1-R, 5**′** –TCCTCTTCTGACTGAGTCATAAG-3**′**; and EWS/FLI1 TaqMan probe, 5**′**-TET-AACAGAGCAGCAGCTACGGGCAGCA-TAMRA-3**′**; For LOX, we used a commercially available TaqMan probe mix (Hs00942480_m1, Applied Biosystems, Foster City, CA); for TATA-binding protein (TBP; used as a reference gene), TBP-F, 5**′**-GAACATCATGGATCAGAACAACAG-3**′** , TBP-R, 5**′** ATTGGTGTTCTGAATAGGCTGTG 3**′**; and TBP TaqMan probe 5**′** FAM-CTGCCACCTTACGCTCAGGGCTTGG-TAMRA 3**′**.

### Western Blot analysis

Whole cell extracts were obtained directly from cell layers lysed in RIPA buffer (1× PBS, 0,1% SDS, 1% NP-40, 0,5% sodium deoxycholate) supplemented with a protease and phosphatase inhibitor cocktail (Roche). For immunoprecipitation of LOX-PP, conditioned media was collected and concentrated 10× using a 10,000 molecular weight cut-off Amicon Ultra centrifugal filter units (Millipore, Billerica, MA, USA). Then, 1.5 ml of concentrated media were cleared with 25 µl of protein A Sepharose (GE Healthcare, Uppsala, Sweden) and supernatant incubated with 1 µl of anti-V5 overnight at 4°C with rocking. Next, 25 µl of protein A Sepharose were added, incubated 1–4 hours at 4°C with rocking and antigen-antibody complex precipitated by centrifugation. The pelleted precipitates containing LOX bound proteins were washed 3 times in PBS containing 0,5% Triton X-100 and 1 mM PMSF and directed resuspended in Laemli buffer. To perform LOX-PP deglycosylation, immunoprecipitated LOX-PP was subjected to Peptide-N-Glycosidase F (PNGase F) treatment according to manufacturer instructions (New England BioLabs, Ipswich, MA). Treated and untreated samples were then resuspended in Laemli buffer prior to gel electrophoresis.

Proteins were subjected to electrophoresis on 10 or 12% SDS-polyacrylamide gel, blotted onto PVDF membranes (Pall, Port Washington, NY, USA) and incubated with the corresponding primary antibodies. After incubation with the secondary HRP-conjugated antibody, membranes were subjected to enhanced chemiluminescence (ECL, GE Healthcare) detection analysis. To ensure equal loading of samples in each lane, membranes were stripped and reprobed with an anti-tubulin monoclonal antibody.

Anti-FLI1 antibody (# 554267) was purchased from BD Pharmingen (San Diego, CA, USA), anti-LOX (# L4794) and anti-tubulin (# T9026) from Sigma-Aldrich, anti-V5 (# R960-25) from Invitrogen, anti-P-Erk (P-Tyr 204, # sc-7383) from Santa Cruz Biotechnology (Santa Cruz, CA, USA), anti-Akt (# 9272), anti-P-Akt (P-Ser 473, # 9271) and anti-Erk (# 9102) from Cell Signalling (Boston, MA, USA). Horseradish peroxidase-conjugated secondary antibodies were purchased from Santa Cruz Biotechonology.

### Cell proliferation assays

Cumulative population doubling was determined in cells grown during 25 days in the absence or in the presence of doxycycline (1 µg/ml) in 100 mm dishes. Before reached confluence, the cells were trypsinized and counted to determine the number of cell duplications. Cell proliferation was also quantified with the CellTiter-Fluor assay (Promega, Madison, WI, USA). Briefly, 3.000 cells were seeded into 96-well plate and allowed to attach for 48 h. Then, cells were incubated in presence of stimulus for 5 days. Culture medium was replaced every 2–3 days with medium containing the corresponding stimulus. At the end of the experiment, CellTiter-Fluor reagent was added to each well and incubated for 1 h at 37°C. Fluorescence was measured using an Infinite M200 (Tecan, Männerdorf, Switzerland) microplate reader. To analyse the effect of an exogenous source of LOX-PP on cell proliferation, we used a standard MTT assay to quantify viable cells (Promega). In these assays, A673 cells were plated at a density of 5.000 cells/well in 96-well plates, incubated during 72 hours with conditioned culture media and then treated with MTT solution according to manufacturer's instructions. Conditioned culture media were obtained and prepared from A673/TR/LOX-PP cells incubated in presence (LOX-PP rich media) or in absence (control media) of doxycycline as described in the previous section.

### Colony formation assay

Cells were plated by triplicate (50,000 cells per 60 mm dishes) in soft agar and cultured in presence or absence of doxycycline during 25 days. Fresh culture medium was added to plates every 2–3 days. At the end of the experiment, three random fields for each plate were photographed. The number of colonies for field and its respective area were calculated using the image analysis software ImageJ [59] (National Institute of Health, Bethesda, MD, USA)

### Cell migration assay

Cells were pre-treated with doxycycline for 24 hours to induce the expression of the corresponding LOX proteins. Next, 300.000 pre-treated cells were suspended in 2 ml of medium containing 0.5% fetal bovine serum and placed in the upper compartment of Transwells (8.0 µm pore size) (Corning Costar, Cambridge, MA, USA). The lower compartment was filled with 3 ml of medium containing 10% fetal bovine serum as chemoattractant. After 6 h of incubation to allow cells to migrate through the membrane, the cells remaining on the upper face of the membrane were removed using a cotton wool swab, while the cells on the lower side of the membrane were fixed with methanol and stained with crystal violet. After washing with PBS, crystal violet was dissolved in PBS with 2% SDS and absorbance quantified at 560 nm using an Infinite M200 (Tecan) microplate reader.

### Tumor formation assay in nude mice

Athymic 6-week-old female BALB/c nu/nu mice (Harlan Ibérica, Barcelona, Spain) were used in these experiments, which were all carried out in accordance with Institutional and European Union guidelines. Cells were washed twice in PBS and resuspended in Matrigel (BD Biosciences) diluted 1/10 in DMEM at a density of 5×10^7^ cells/ml. Animals were subcutaneously injected with the cell solution (0.1 ml) into the left flanks of the mice. Animals were then kept under pathogen free conditions and observed daily for any visible signs of tumors at the injection sites. The tumor volume was measured every 2 or 3 days and calculated using the formula L×W^2^×π/6, where L is the length and W is the width of the tumor. When indicated, doxycycline was given by oral route in natural mineral water at a concentration of 1 mg/mL Control animals received water alone. When tumor volume reached 0,5 cm^3^, mice were sacrificed and tumors were removed.

### Gene expression profiles and functional genomic analyses

To identify genes and pathways regulated by LOX-PP in A673 cells, we used Agilent SurePrint G3 60K v2 microarrays. We analysed the RNA isolated from three separate experiments performed with A673/TR/empty (control cells) and A673/TR/LOX-PP cells stimulated with doxycycline for 72 h to induce the expression of LOX-PP. Labelling, hybridization and scanning were performed at NIMGenetics (Madrid, Spain). Functional genomic analysis was carried out using Gene Set Enrichment Analyses (GSEA) (http://www.broadinstitute.org/gsea/index.jsp, [42]. GSEA is a computational method that determines whether an a priori defined set of genes (made for example by genes that belong to a defined pathway or by genes that share a cis-regulatory motif) is overrepresented at the top or bottom of a ranked list of genes (in our case the list of genes differentially regulated by LOX-PP, ranked using the Student's t-test metric). Gene set permutation was carried out to compute NES (Normalized Enrichment Score) that reflects the degree to which a gene set is overrepresented at the top or bottom of a ranked list of genes. We considered that a gene set/pathway is significantly altered by LOX-PP if that gene set reached a False Discovery Rate (FDR) bellow 0.5% (FDR<0.005), which represent a very stringent FDR cut-off. The FDR estimates the probability that a gene set with a given NES represents a false positive finding.

### Statistical analysis

For a single comparison of two groups, two tailed Student's t test was used. For all analyses, the level of significance was set at *P*<0.05. All statistical calculations were made using the GraphPad Prism statistical software version 4.0 (GraphPad Software, San Diego, CA).

## Supporting Information

Figure S1 **Relative expression of LOX in human tumors.**
(TIFF)

Figure S2 **Functional analysis of gene expression profiles deregulated by LOX-PP (GSEA analysis).**
(TIFF)

Table S1 **Summary report of gene sets derived from the Reactome pathway database enrichment in A673 cells upon LOX-PP expression.**
(PDF)
