# Supplementary figures and images for: Lysyl Oxidase Is Downregulated by the EWS/FLI1 Oncoprotein and Its Propeptide Domain Displays Tumor Supressor Activities in Ewing Sarcoma Cells
Source: PLoS One. 2013 Jun 4;8(6):e66281. doi: 10.1371/journal.pone.0066281 (PMC3672102; doi:10.1371/journal.pone.0066281)

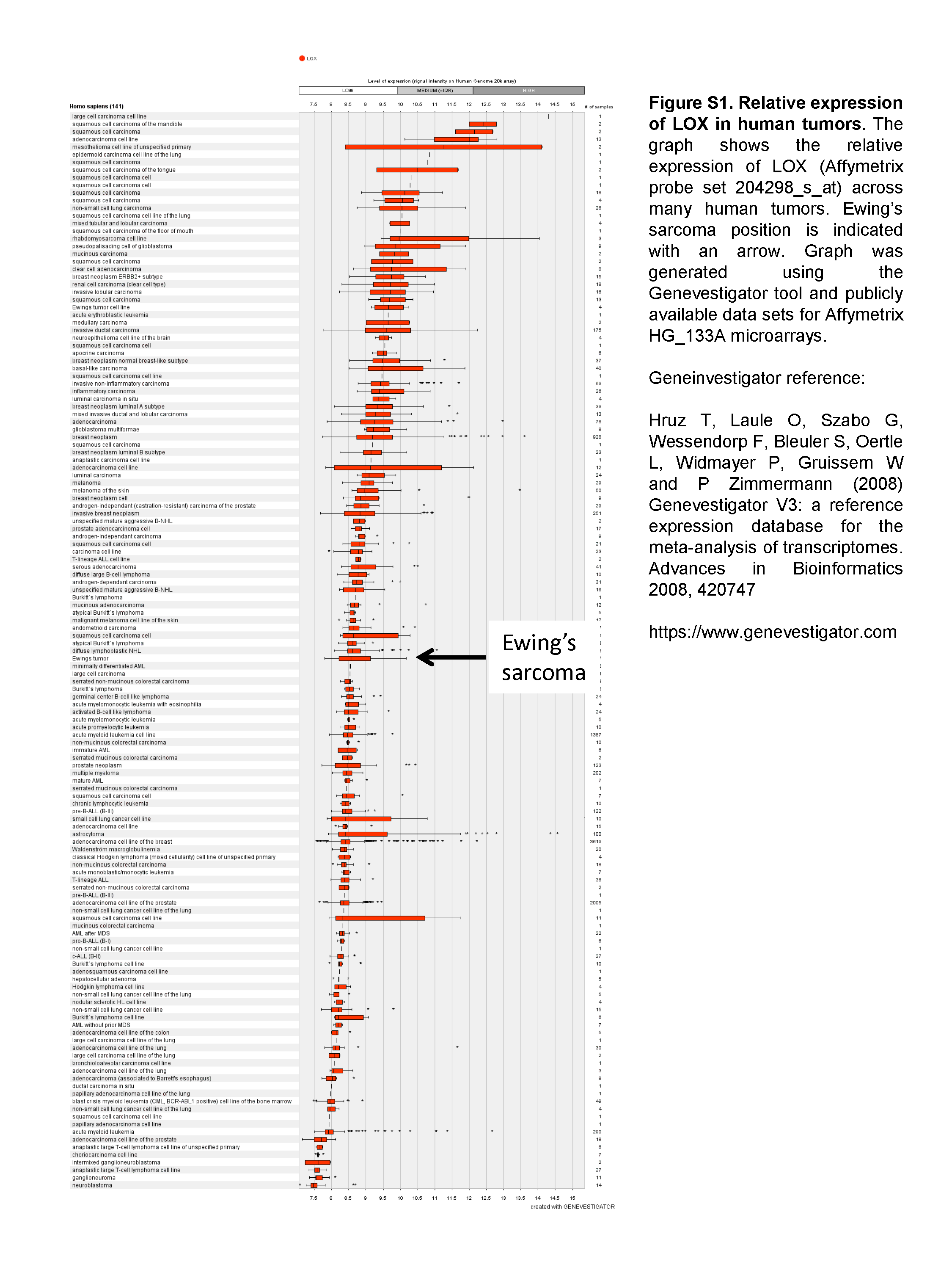

Supplement: Figure S1 — Relative expression of LOX in human tumors. (TIFF) [file pone.0066281.s001.tiff]

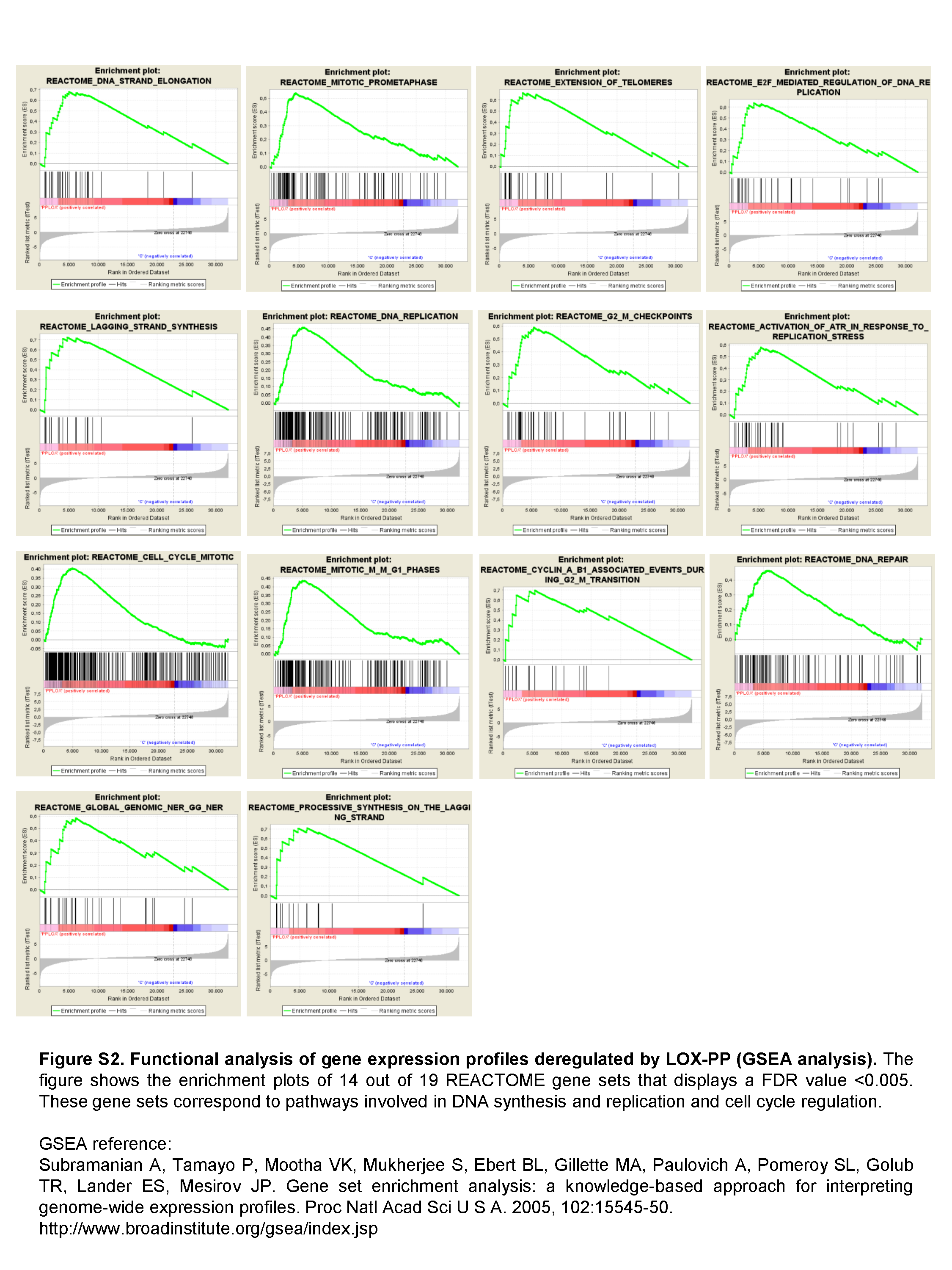

Supplement: Figure S2 — Functional analysis of gene expression profiles deregulated by LOX-PP (GSEA analysis). (TIFF) [file pone.0066281.s002.tiff]
