# Supplementary material for: Lysyl Oxidase Is Downregulated by the EWS/FLI1 Oncoprotein and Its Propeptide Domain Displays Tumor Supressor Activities in Ewing Sarcoma Cells
Source: PLoS One. 2013 Jun 4;8(6):e66281. doi: 10.1371/journal.pone.0066281 (PMC3672102; doi:10.1371/journal.pone.0066281)
Supplement: Table S1 — Summary report of gene sets derived from the Reactome pathway database enrichment in A673 cells upon LOX-PP expression. (PDF) [file pone.0066281.s003.pdf]

Table S1. Summary report of gene sets derived from the Reactome pathway database enrichment in A673 cells upon LOX-PP expression.

	GENE SET (follow link to MSigDB)	SIZE	ES	NES	NOM p-val	FDR q-val	FWER p-val	RANK AT MAX
1	REACTOME_CHOLESTEROL_BIOSYNTHESIS	22	0,84	2,83	0	0	0	3974
2	REACTOME_DNA_STRAND_ELONGATION	30	0,68	2,45	0	0	0	4974
3	REACTOME_MITOTIC_PROMETAPHASE	86	0,54	2,42	0	0	0	4434
4	REACTOME_EXTENSION_OF_TELOMERES	27	0,66	2,3	0	0,002	0,003	3955
5	REACTOME_E2F_MEDIATED_REGULATION_OF_DNA_REPLICATION	33	0,64	2,29	0	0,001	0,003	4168
6	REACTOME_COLLAGEN_FORMATION	58	0,57	2,29	0	0,001	0,003	2809
7	REACTOME_LAGGING_STRAND_SYNTHESIS	19	0,72	2,27	0	0,001	0,004	4722
8	REACTOME_DNA_REPLICATION	188	0,46	2,27	0	0,001	0,004	4974
9	REACTOME_G2_M_CHECKPOINTS	41	0,59	2,23	0	0,002	0,007	5346
10	REACTOME_EXTRACELLULAR_MATRIX_ORGANIZATION	87	0,49	2,2	0	0,001	0,007	2809
11	REACTOME_ACTIVATION_OF_ATR_IN_RESPONSE_TO_REPLICATION_STRESS	35	0,58	2,17	0	0,002	0,012	5346
12	REACTOME_CITRIC_ACID_CYCLE_TCA_CYCLE	19	0,69	2,17	0	0,002	0,014	3904
13	REACTOME_CELL_CYCLE_MITOTIC	308	0,41	2,15	0	0,003	0,016	4974
14	REACTOME_MITOTIC_M_M_G1_PHASES	168	0,44	2,14	0	0,003	0,018	5316
15	REACTOME_FATTY_ACID_TRIACYLGLYCEROL_AND_KETONE_BODY_METABOLISM	167	0,42	2,11	0	0,004	0,029	3626
16	REACTOME_CYCLIN_A_B1_ASSOCIATED_EVENTS_DURING_G2_M_TRANSITION	15	0,7	2,1	0,003	0,005	0,036	5416
17	REACTOME_DNA_REPAIR	101	0,46	2,1	0	0,004	0,036	5508
18	REACTOME_GLOBAL_GENOMIC_NER_GG_NER	31	0,58	2,07	0	0,005	0,045	6189
19	REACTOME_PROCESSIVE_SYNTHESIS_ON_THE_LAGGING_STRAND	15	0,71	2,06	0	0,005	0,047	6261

Comments

19 gene sets were significant enrichment at FDR<0.005.

Among then, 14 were related to DNA synthesis and replication and cell cycle regulation: DNA strand elongation, Mitotic prometaphase, Extension of telomeres, E2F mediated regulation of DNA replication, Lagging strand synthesis, DNA replication, G2/M checkpoints, Activation of ATR in response to replication stress, Cell cycle mitotic, Mitotic M and M/G1 phases, Cyclin A/B1 associated events during G2/M transition, DNA repair, Global Genomic Non-Excision-Repair, Processive syntesis on the lagging strand.

3 gene sets were related to cell metabolism: Cholesterol Biosynthesis, Citric acid cycle, Fatty acid triacylglycerol and ketone body metabolism.

2 gene sets were related to extracellular matrix: Collagen formation, Extracellular matrix organization

Click on the gene set name to go to descriptions at the GSEA home page. Column description is provided in the page 2 of this document.

Lysyl oxidase is downregulated by the EWS/FLI1 oncoprotein and its propeptide domain displays tumor supresor activities in Ewing sarcoma cells.
Agra N. et al.

Column descriptions

GENE SET: Gene set name. Click the gene set name for a detailed description of the gene set. For MSigDB gene sets, the description is the gene set page on the GSEA web site. For other gene sets, the description is provided by the author of the gene set.

SIZE: Number of genes in the gene set after filtering out those genes not in the expression dataset

ES: Enrichment score for the gene set; that is, the degree to which this gene set is overrepresented at the top or bottom of the ranked list of genes in the expression dataset.

NES: Normalized enrichment score; that is, the enrichment score for the gene set after it has been normalized across analyzed gene sets.

NOM p-value: Nominal p value; that is, the statistical significance of the enrichment score. The nominal p value is not adjusted for gene set size or multiple hypothesis testing; therefore, it is of limited use in comparing gene sets.

FDR q-value: False discovery rate; that is, the estimated probability that the normalized enrichment score represents a false positive finding.

FWER p-value: Familywise-error rate; that is, a more conservatively estimated probability that the normalized enrichment score represents a false positive finding. Because the goal of GSEA is to generate hypotheses, the GSEA team recommends focusing on the FDR statistic.

RANK AT MAX: The position in the ranked list at which the maximum enrichment score occurred. The more interesting gene sets achieve the maximum
